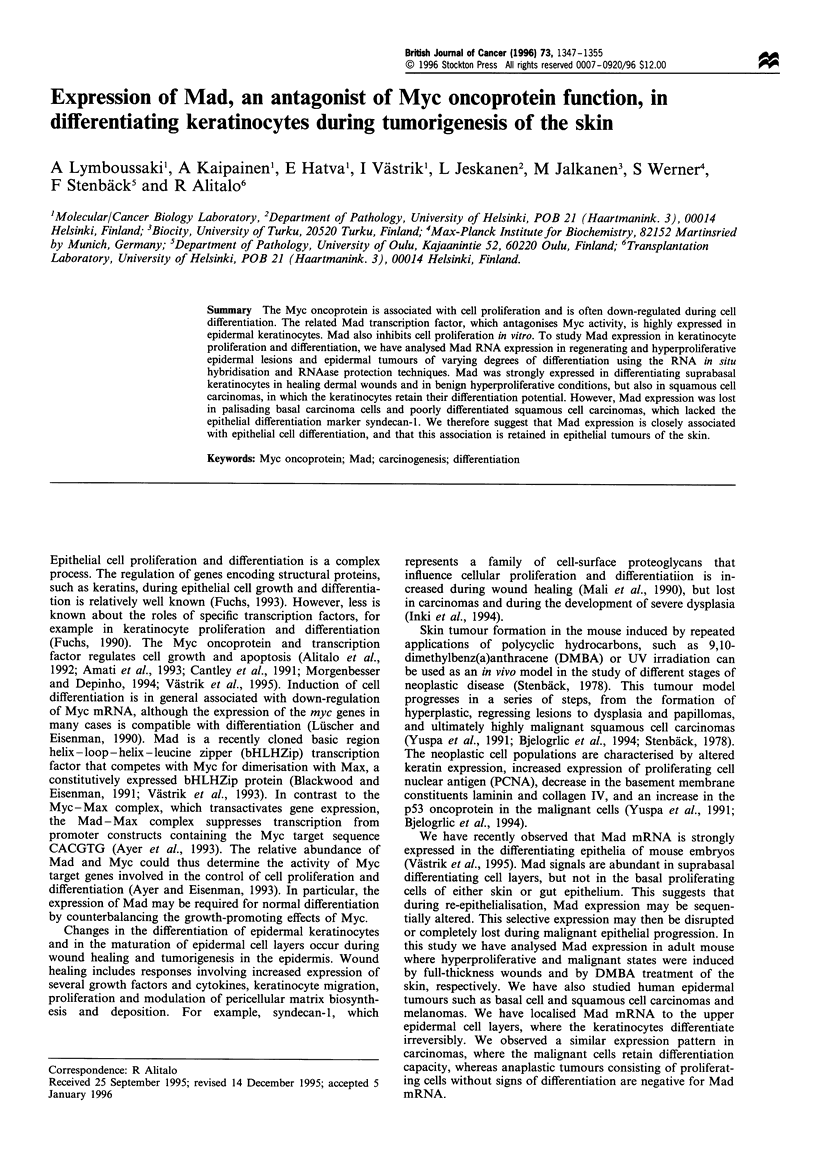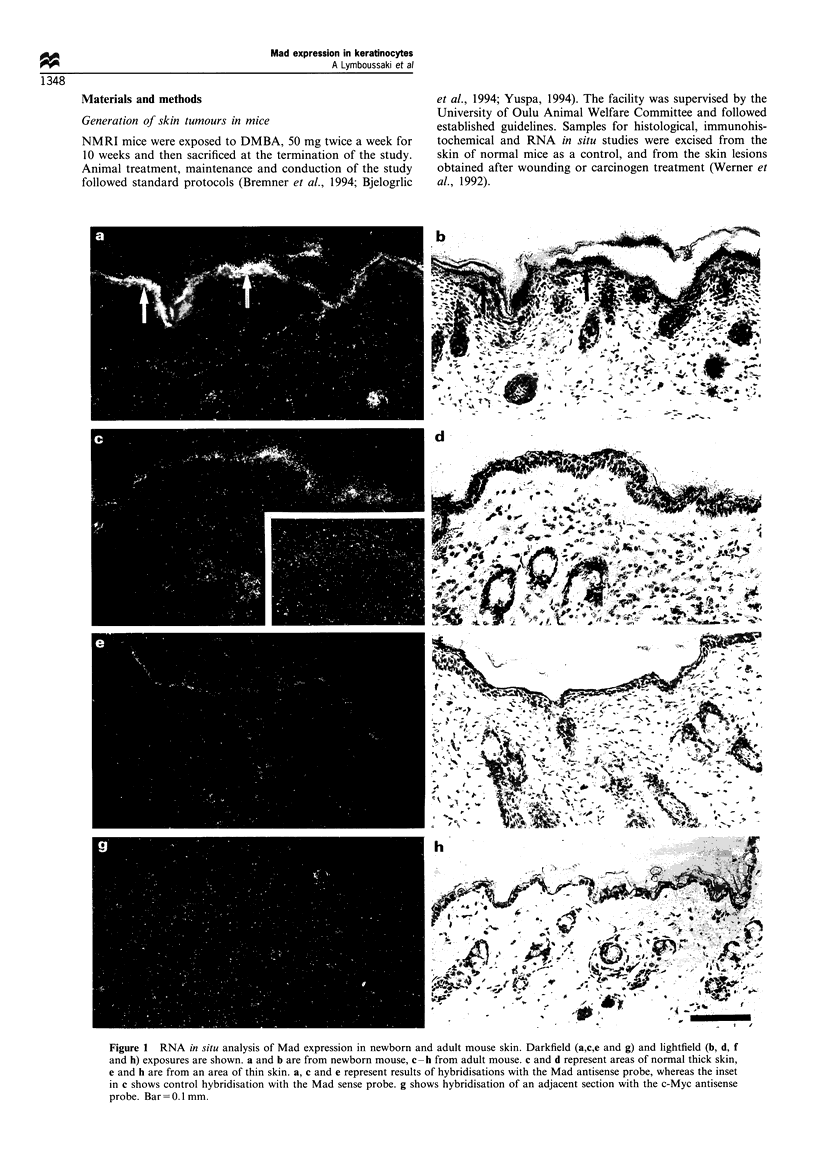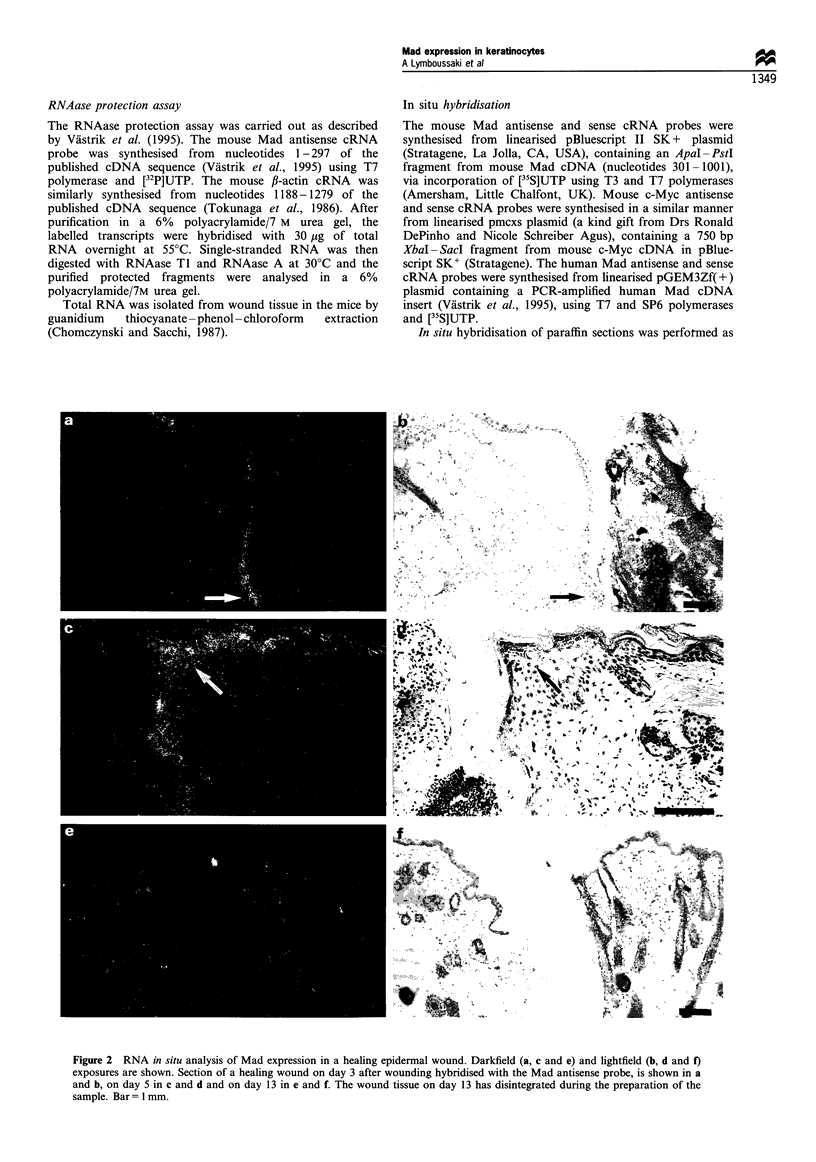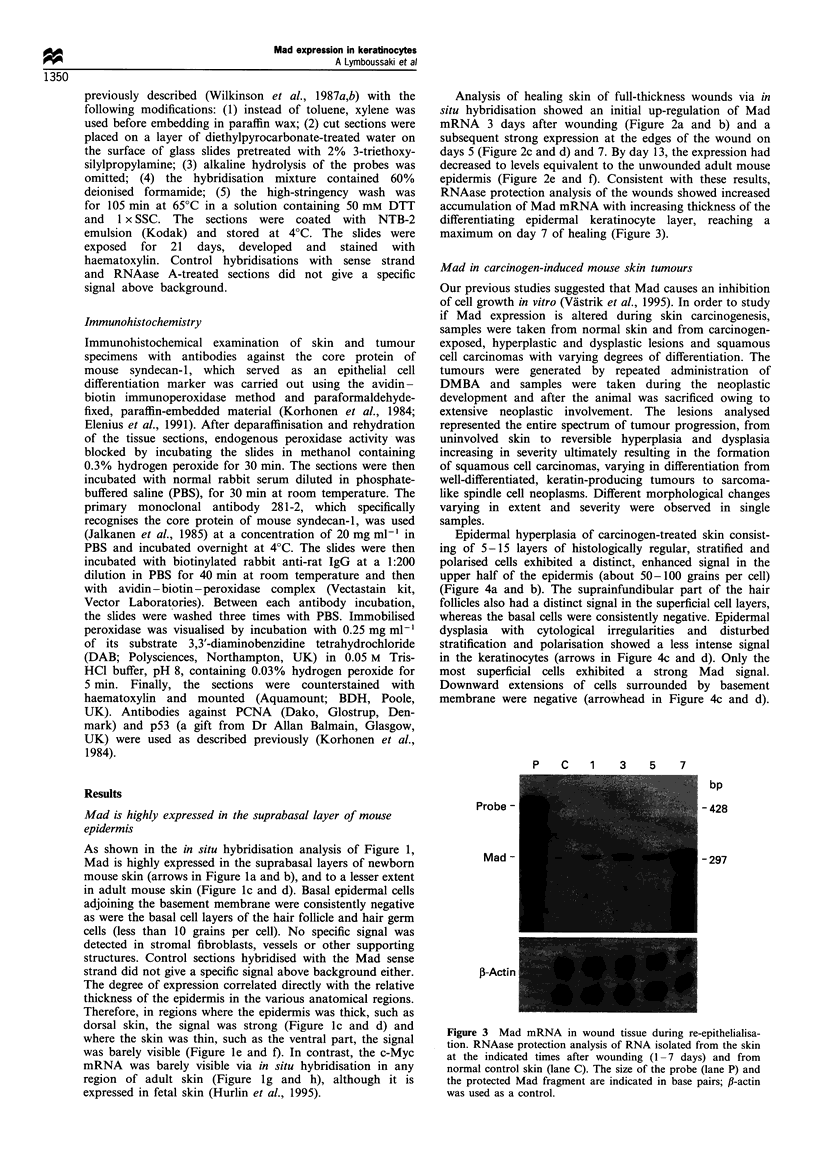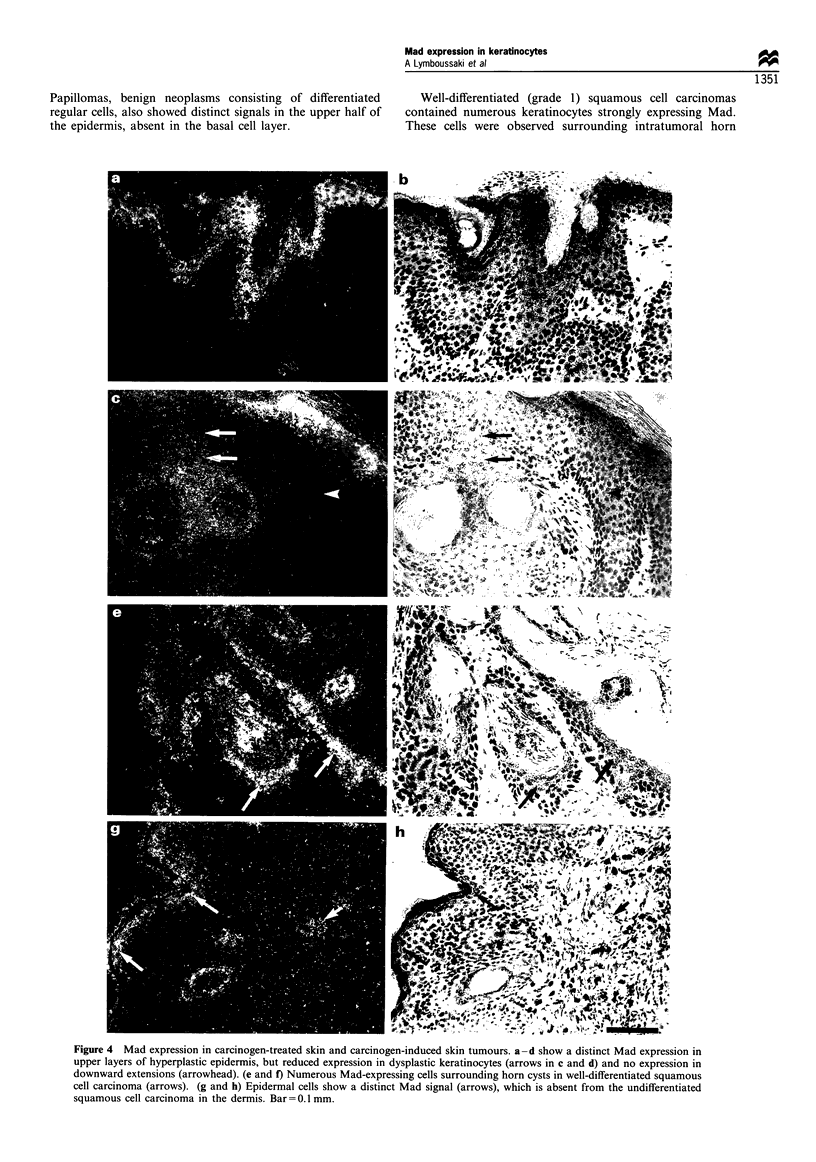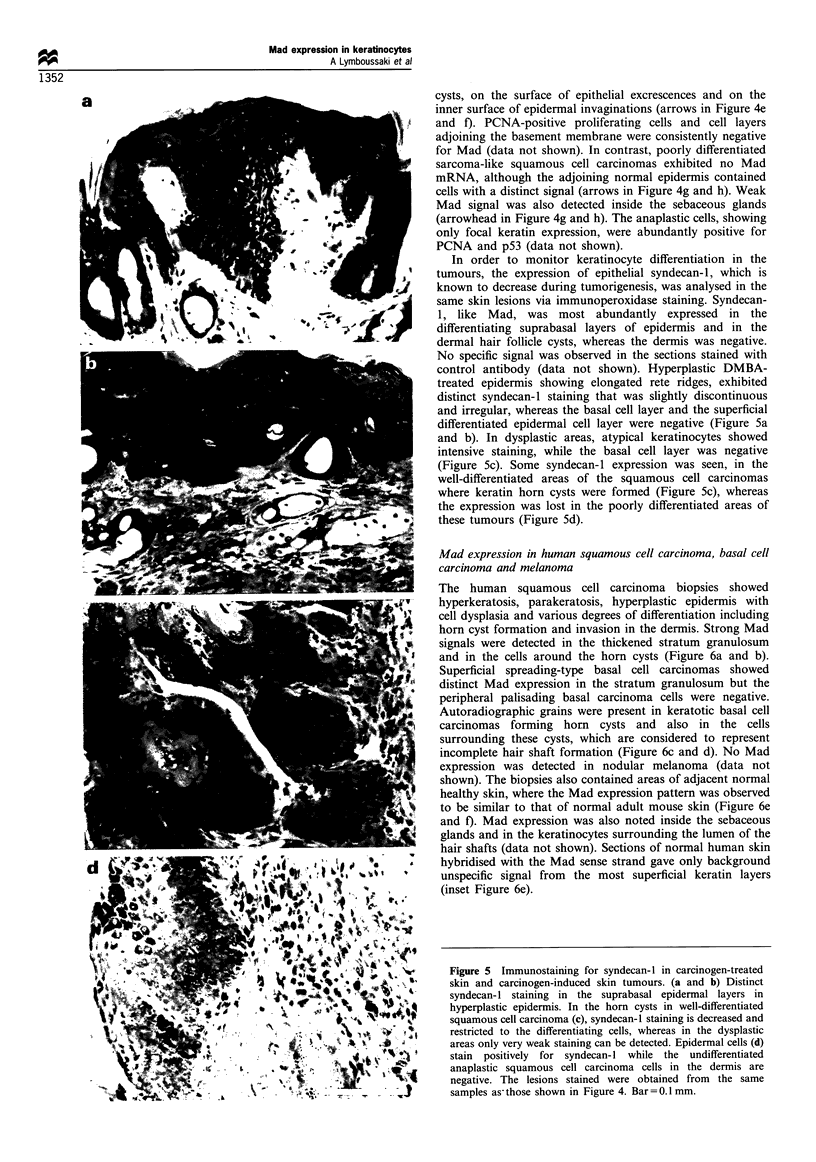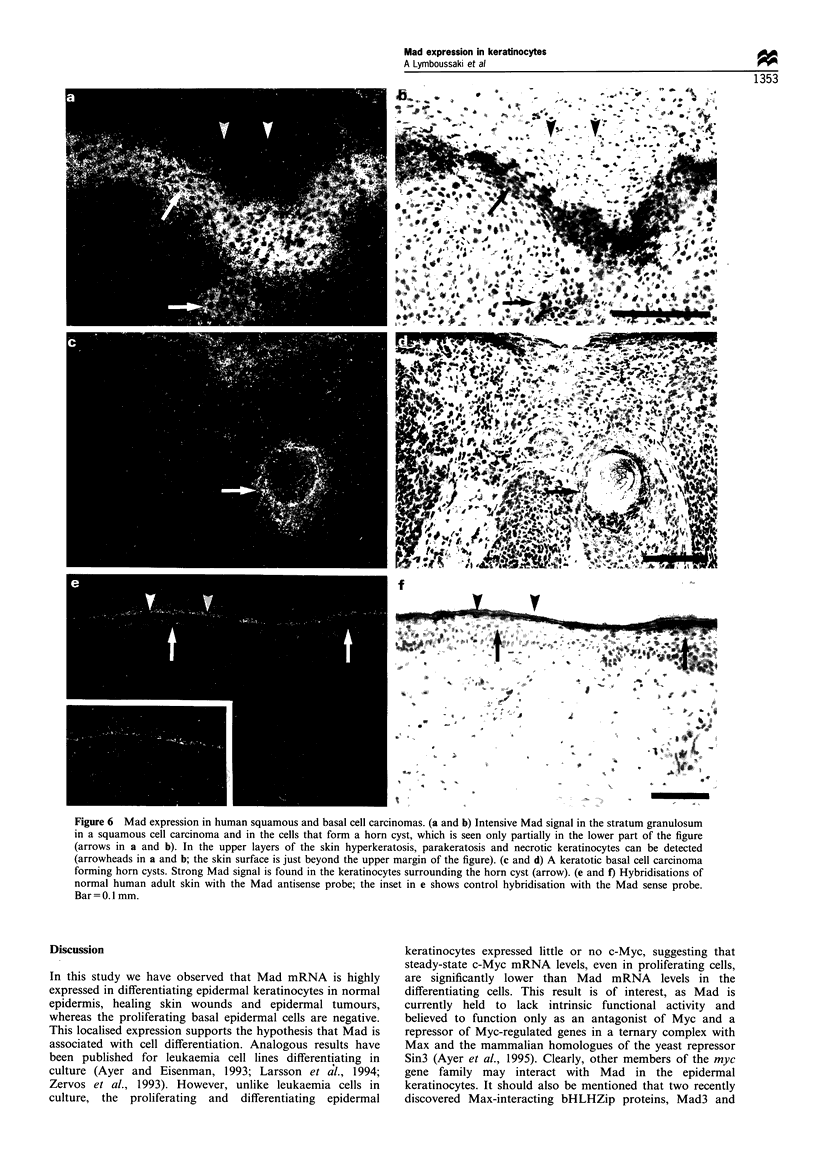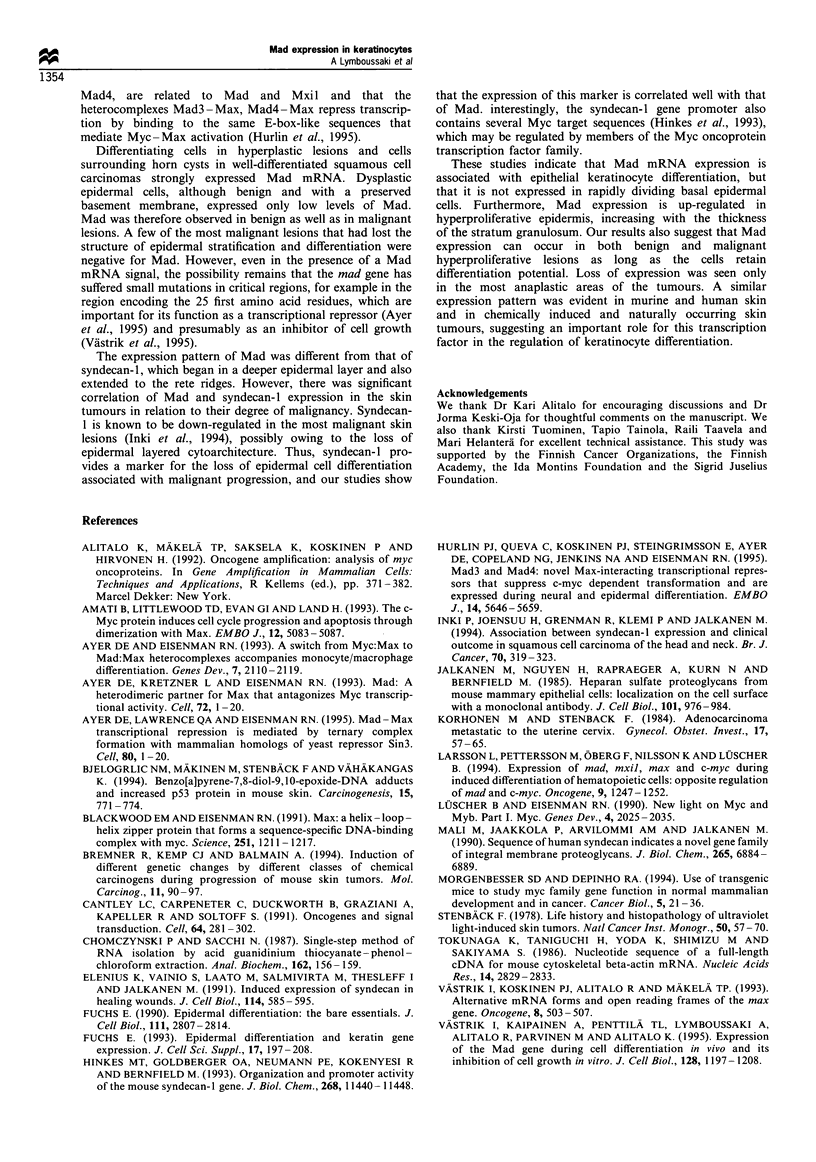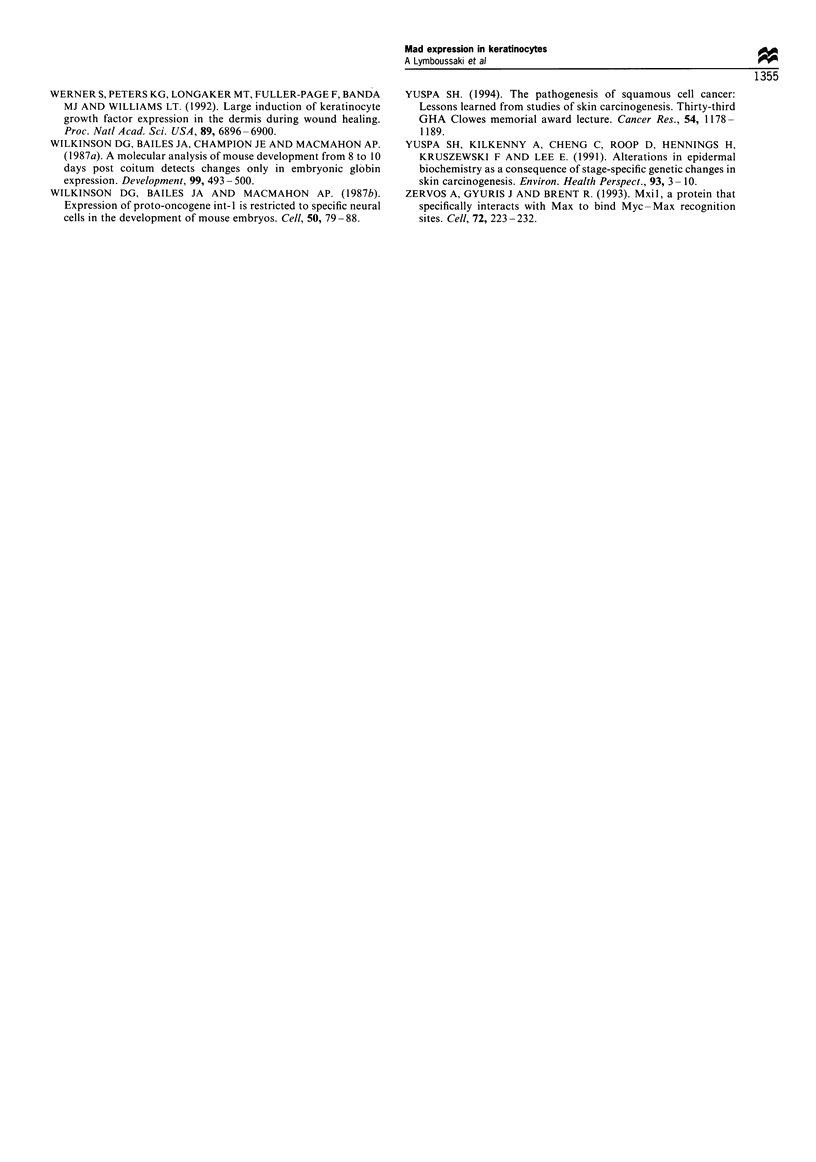# Expression of Mad, an antagonist of Myc oncoprotein function, in differentiating keratinocytes during tumorigenesis of the skin.

**DOI:** 10.1038/bjc.1996.257

**Published:** 1996-06

**Authors:** A. Lymboussaki, A. Kaipainen, E. Hatva, I. Västrik, L. Jeskanen, M. Jalkanen, S. Werner, F. Stenbäck, R. Alitalo

**Affiliations:** Molecular/Cancer Biology Laboratory, University of Helsinki, Finland.

## Abstract

**Images:**


British Journal of Cancer (1996) 73, 1347-1355
© 1996 Stockton Press All rights reserved 0007-0920/96 $12.00

### Expression of Mad, an antagonist of Myc oncoprotein function, in differentiating keratinocytes during tumorigenesis of the skin

A Lymboussaki[1], A Kaipainen[1], E Hatva[1], I Västrik[1], L Jeskanen[2], M Jalkanen[3], S Werner[4], F Stenbäck[5] and R Alitalo[6]

*[1]Molecular/Cancer Biology Laboratory, [2]Department of Pathology, University of Helsinki, POB 21 (Haartmanink. 3), 00014 Helsinki, Finland; [3]Biocity, University of Turku, 20520 Turku, Finland; [4]Max-Planck Institute for Biochemistry, 82152 Martinsried by Munich, Germany; [5]Department of Pathology, University of Oulu, Kajaanintie 52, 60220 Oulu, Finland; [6]Transplantation Laboratory, University of Helsinki, POB 21 (Haartmanink. 3), 00014 Helsinki, Finland.*

**Summary** The Myc oncoprotein is associated with cell proliferation and is often down-regulated during cell differentiation. The related Mad transcription factor, which antagonises Myc activity, is highly expressed in epidermal keratinocytes. Mad also inhibits cell proliferation *in vitro*. To study Mad expression in keratinocyte proliferation and differentiation, we have analysed Mad RNA expression in regenerating and hyperproliferative epidermal lesions and epidermal tumours of varying degrees of differentiation using the RNA *in situ* hybridisation and RNAase protection techniques. Mad was strongly expressed in differentiating suprabasal keratinocytes in healing dermal wounds and in benign hyperproliferative conditions, but also in squamous cell carcinomas, in which the keratinocytes retain their differentiation potential. However, Mad expression was lost in palisading basal carcinoma cells and poorly differentiated squamous cell carcinomas, which lacked the epithelial differentiation marker syndecan-1. We therefore suggest that Mad expression is closely associated with epithelial cell differentiation, and that this association is retained in epithelial tumours of the skin.

**Keywords:** Myc oncoprotein; Mad; carcinogenesis; differentiation

Epithelial cell proliferation and differentiation is a complex process. The regulation of genes encoding structural proteins, such as keratins, during epithelial cell growth and differentiation is relatively well known (Fuchs, 1993). However, less is known about the roles of specific transcription factors, for example in keratinocyte proliferation and differentiation (Fuchs, 1990). The Myc oncoprotein and transcription factor regulates cell growth and apoptosis (Alitalo *et al.*, 1992; Amati *et al.*, 1993; Cantley *et al.*, 1991; Morgenbesser and Depinho, 1994; Västrik *et al.*, 1995). Induction of cell differentiation is in general associated with down-regulation of Myc mRNA, although the expression of the *myc* genes in many cases is compatible with differentiation (Lüscher and Eisenman, 1990). Mad is a recently cloned basic region helix-loop-helix-leucine zipper (bHLHZip) transcription factor that competes with Myc for dimerisation with Max, a constitutively expressed bHLHZip protein (Blackwood and Eisenman, 1991; Västrik *et al.*, 1993). In contrast to the Myc-Max complex, which transactivates gene expression, the Mad-Max complex suppresses transcription from promoter constructs containing the Myc target sequence CACGTG (Ayer *et al.*, 1993). The relative abundance of Mad and Myc could thus determine the activity of Myc target genes involved in the control of cell proliferation and differentiation (Ayer and Eisenman, 1993). In particular, the expression of Mad may be required for normal differentiation by counterbalancing the growth-promoting effects of Myc.

Changes in the differentiation of epidermal keratinocytes and in the maturation of epidermal cell layers occur during wound healing and tumorigenesis in the epidermis. Wound healing includes responses involving increased expression of several growth factors and cytokines, keratinocyte migration, proliferation and modulation of pericellular matrix biosynthesis and deposition. For example, syndecan-1, which represents a family of cell-surface proteoglycans that influence cellular proliferation and differentiatiion is increased during wound healing (Mali *et al.*, 1990), but lost in carcinomas and during the development of severe dysplasia (Inki *et al.*, 1994).

Skin tumour formation in the mouse induced by repeated applications of polycyclic hydrocarbons, such as 9,10-dimethylbenz(a)anthracene (DMBA) or UV irradiation can be used as an *in vivo* model in the study of different stages of neoplastic disease (Stenbäck, 1978). This tumour model progresses in a series of steps, from the formation of hyperplastic, regressing lesions to dysplasia and papillomas, and ultimately highly malignant squamous cell carcinomas (Yuspa *et al.*, 1991; Bjelogrlic *et al.*, 1994; Stenbäck, 1978). The neoplastic cell populations are characterised by altered keratin expression, increased expression of proliferating cell nuclear antigen (PCNA), decrease in the basement membrane constituents laminin and collagen IV, and an increase in the p53 oncoprotein in the malignant cells (Yuspa *et al.*, 1991; Bjelogrlic *et al.*, 1994).

We have recently observed that Mad mRNA is strongly expressed in the differentiating epithelia of mouse embryos (Västrik *et al.*, 1995). Mad signals are abundant in suprabasal differentiating cell layers, but not in the basal proliferating cells of either skin or gut epithelium. This suggests that during re-epithelialisation, Mad expression may be sequentially altered. This selective expression may then be disrupted or completely lost during malignant epithelial progression. In this study we have analysed Mad expression in adult mouse where hyperproliferative and malignant states were induced by full-thickness wounds and by DMBA treatment of the skin, respectively. We have also studied human epidermal tumours such as basal cell and squamous cell carcinomas and melanomas. We have localised Mad mRNA to the upper epidermal cell layers, where the keratinocytes differentiate irreversibly. We observed a similar expression pattern in carcinomas, where the malignant cells retain differentiation capacity, whereas anaplastic tumours consisting of proliferating cells without signs of differentiation are negative for Mad mRNA.

Correspondence: R Alitalo
Received 25 September 1995; revised 14 December 1995; accepted 5 January 1996

### Materials and methods

*Generation of skin tumours in mice*

NMRI mice were exposed to DMBA, 50 mg twice a week for 10 weeks and then sacrificed at the termination of the study. Animal treatment, maintenance and conduction of the study followed standard protocols (Bremner *et al.*, 1994; Bjelogrlic *et al.*, 1994; Yuspa, 1994). The facility was supervised by the University of Oulu Animal Welfare Committee and followed established guidelines. Samples for histological, immunohistochemical and RNA *in situ* studies were excised from the skin of normal mice as a control, and from the skin lesions obtained after wounding or carcinogen treatment (Werner *et al.*, 1992).

**Figure 1** RNA *in situ* analysis of Mad expression in newborn and adult mouse skin. Darkfield (**a**,**c**,**e** and **g**) and lightfield (**b**, **d**, **f** and **h**) exposures are shown. **a** and **b** are from newborn mouse, **c**–**h** from adult mouse. **c** and **d** represent areas of normal thick skin, **e** and **h** are from an area of thin skin. **a**, **c** and **e** represent results of hybridisations with the Mad antisense probe, whereas the inset in **c** shows control hybridisation with the Mad sense probe. **g** shows hybridisation of an adjacent section with the c-Myc antisense probe. Bar = 0.1 mm.

*RNAase protection assay*

The RNAase protection assay was carried out as described by Västrik *et al.* (1995). The mouse Mad antisense cRNA probe was synthesised from nucleotides 1–297 of the published cDNA sequence (Västrik *et al.*, 1995) using T7 polymerase and [$^{32}$P]UTP. The mouse $\beta$-actin cRNA was similarly synthesised from nucleotides 1188–1279 of the published cDNA sequence (Tokunaga *et al.*, 1986). After purification in a 6% polyacrylamide/7 M urea gel, the labelled transcripts were hybridised with 30 $\mu$g of total RNA overnight at 55°C. Single-stranded RNA was then digested with RNAase T1 and RNAase A at 30°C and the purified protected fragments were analysed in a 6% polyacrylamide/7M urea gel.

Total RNA was isolated from wound tissue in the mice by guanidium thiocyanate–phenol–chloroform extraction (Chomczynski and Sacchi, 1987).

In situ *hybridisation*

The mouse Mad antisense and sense cRNA probes were synthesised from linearised pBluescript II SK+ plasmid (Stratagene, La Jolla, CA, USA), containing an *Apa*I–*Pst*I fragment from mouse Mad cDNA (nucleotides 301–1001), via incorporation of [$^{35}$S]UTP using T3 and T7 polymerases (Amersham, Little Chalfont, UK). Mouse c-Myc antisense and sense cRNA probes were synthesised in a similar manner from linearised pmcxs plasmid (a kind gift from Drs Ronald DePinho and Nicole Schreiber Agus), containing a 750 bp *Xba*I–*Sac*I fragment from mouse c-Myc cDNA in pBluescript SK$^{+}$ (Stratagene). The human Mad antisense and sense cRNA probes were synthesised from linearised pGEM3Zf(+) plasmid containing a PCR-amplified human Mad cDNA insert (Västrik *et al.*, 1995), using T7 and SP6 polymerases and [$^{35}$S]UTP.

*In situ* hybridisation of paraffin sections was performed as

**Figure 2** RNA *in situ* analysis of Mad expression in a healing epidermal wound. Darkfield (**a**, **c** and **e**) and lightfield (**b**, **d** and **f**) exposures are shown. Section of a healing wound on day 3 after wounding hybridised with the Mad antisense probe, is shown in **a** and **b**, on day 5 in **c** and **d** and on day 13 in **e** and **f**. The wound tissue on day 13 has disintegrated during the preparation of the sample. Bar = 1 mm.

previously described (Wilkinson *et al.*, 1987*a,b*) with the following modifications: (1) instead of toluene, xylene was used before embedding in paraffin wax; (2) cut sections were placed on a layer of diethylpyrocarbonate-treated water on the surface of glass slides pretreated with 2% 3-triethoxy-silylpropylamine; (3) alkaline hydrolysis of the probes was omitted; (4) the hybridisation mixture contained 60% deionised formamide; (5) the high-stringency wash was for 105 min at 65°C in a solution containing 50 mM DTT and 1 × SSC. The sections were coated with NTB-2 emulsion (Kodak) and stored at 4°C. The slides were exposed for 21 days, developed and stained with haematoxylin. Control hybridisations with sense strand and RNAase A-treated sections did not give a specific signal above background.

*Immunohistochemistry*

Immunohistochemical examination of skin and tumour specimens with antibodies against the core protein of mouse syndecan-1, which served as an epithelial cell differentiation marker was carried out using the avidin–biotin immunoperoxidase method and paraformaldehyde-fixed, paraffin-embedded material (Korhonen *et al.*, 1984; Elenius *et al.*, 1991). After deparaffinisation and rehydration of the tissue sections, endogenous peroxidase activity was blocked by incubating the slides in methanol containing 0.3% hydrogen peroxide for 30 min. The sections were then incubated with normal rabbit serum diluted in phosphate-buffered saline (PBS), for 30 min at room temperature. The primary monoclonal antibody 281-2, which specifically recognises the core protein of mouse syndecan-1, was used (Jalkanen *et al.*, 1985) at a concentration of 20 mg $ml^{-1}$ in PBS and incubated overnight at 4°C. The slides were then incubated with biotinylated rabbit anti-rat IgG at a 1:200 dilution in PBS for 40 min at room temperature and then with avidin–biotin–peroxidase complex (Vectastain kit, Vector Laboratories). Between each antibody incubation, the slides were washed three times with PBS. Immobilised peroxidase was visualised by incubation with 0.25 mg $ml^{-1}$ of its substrate 3,3′-diaminobenzidine tetrahydrochloride (DAB; Polysciences, Northampton, UK) in 0.05 M Tris-HCl buffer, pH 8, containing 0.03% hydrogen peroxide for 5 min. Finally, the sections were counterstained with haematoxylin and mounted (Aquamount; BDH, Poole, UK). Antibodies against PCNA (Dako, Glostrup, Denmark) and p53 (a gift from Dr Allan Balmain, Glasgow, UK) were used as described previously (Korhonen *et al.*, 1984).

### Results

*Mad is highly expressed in the suprabasal layer of mouse epidermis*

As shown in the *in situ* hybridisation analysis of Figure 1, Mad is highly expressed in the suprabasal layers of newborn mouse skin (arrows in Figure 1a and b), and to a lesser extent in adult mouse skin (Figure 1c and d). Basal epidermal cells adjoining the basement membrane were consistently negative as were the basal cell layers of the hair follicle and hair germ cells (less than 10 grains per cell). No specific signal was detected in stromal fibroblasts, vessels or other supporting structures. Control sections hybridised with the Mad sense strand did not give a specific signal above background either. The degree of expression correlated directly with the relative thickness of the epidermis in the various anatomical regions. Therefore, in regions where the epidermis was thick, such as dorsal skin, the signal was strong (Figure 1c and d) and where the skin was thin, such as the ventral part, the signal was barely visible (Figure 1e and f). In contrast, the c-Myc mRNA was barely visible via *in situ* hybridisation in any region of adult skin (Figure 1g and h), although it is expressed in fetal skin (Hurlin *et al.*, 1995).

Analysis of healing skin of full-thickness wounds via *in situ* hybridisation showed an initial up-regulation of Mad mRNA 3 days after wounding (Figure 2a and b) and a subsequent strong expression at the edges of the wound on days 5 (Figure 2c and d) and 7. By day 13, the expression had decreased to levels equivalent to the unwounded adult mouse epidermis (Figure 2e and f). Consistent with these results, RNAase protection analysis of the wounds showed increased accumulation of Mad mRNA with increasing thickness of the differentiating epidermal keratinocyte layer, reaching a maximum on day 7 of healing (Figure 3).

*Mad in carcinogen-induced mouse skin tumours*

Our previous studies suggested that Mad causes an inhibition of cell growth *in vitro* (Västrik *et al.*, 1995). In order to study if Mad expression is altered during skin carcinogenesis, samples were taken from normal skin and from carcinogen-exposed, hyperplastic and dysplastic lesions and squamous cell carcinomas with varying degrees of differentiation. The tumours were generated by repeated administration of DMBA and samples were taken during the neoplastic development and after the animal was sacrificed owing to extensive neoplastic involvement. The lesions analysed represented the entire spectrum of tumour progression, from uninvolved skin to reversible hyperplasia and dysplasia increasing in severity ultimately resulting in the formation of squamous cell carcinomas, varying in differentiation from well-differentiated, keratin-producing tumours to sarcoma-like spindle cell neoplasms. Different morphological changes varying in extent and severity were observed in single samples.

Epidermal hyperplasia of carcinogen-treated skin consisting of 5–15 layers of histologically regular, stratified and polarised cells exhibited a distinct, enhanced signal in the upper half of the epidermis (about 50–100 grains per cell) (Figure 4a and b). The suprainfundibular part of the hair follicles also had a distinct signal in the superficial cell layers, whereas the basal cells were consistently negative. Epidermal dysplasia with cytological irregularities and disturbed stratification and polarisation showed a less intense signal in the keratinocytes (arrows in Figure 4c and d). Only the most superficial cells exhibited a strong Mad signal. Downward extensions of cells surrounded by basement membrane were negative (arrowhead in Figure 4c and d).

**Figure 3** Mad mRNA in wound tissue during re-epithelialisation. RNAase protection analysis of RNA isolated from the skin at the indicated times after wounding (1–7 days) and from normal control skin (lane C). The size of the probe (lane P) and the protected Mad fragment are indicated in base pairs; *β*-actin was used as a control.

Papillomas, benign neoplasms consisting of differentiated regular cells, also showed distinct signals in the upper half of the epidermis, absent in the basal cell layer.

Well-differentiated (grade 1) squamous cell carcinomas contained numerous keratinocytes strongly expressing Mad. These cells were observed surrounding intratumoral horn

**Figure 4** Mad expression in carcinogen-treated skin and carcinogen-induced skin tumours. **a**–**d** show a distinct Mad expression in upper layers of hyperplastic epidermis, but reduced expression in dysplastic keratinocytes (arrows in **c** and **d**) and no expression in downward extensions (arrowhead). (**e** and **f**) Numerous Mad-expressing cells surrounding horn cysts in well-differentiated squamous cell carcinoma (arrows). (**g** and **h**) Epidermal cells show a distinct Mad signal (arrows), which is absent from the undifferentiated squamous cell carcinoma in the dermis. Bar = 0.1 mm.

cysts, on the surface of epithelial excrescences and on the inner surface of epidermal invaginations (arrows in Figure 4e and f). PCNA-positive proliferating cells and cell layers adjoining the basement membrane were consistently negative for Mad (data not shown). In contrast, poorly differentiated sarcoma-like squamous cell carcinomas exhibited no Mad mRNA, although the adjoining normal epidermis contained cells with a distinct signal (arrows in Figure 4g and h). Weak Mad signal was also detected inside the sebaceous glands (arrowhead in Figure 4g and h). The anaplastic cells, showing only focal keratin expression, were abundantly positive for PCNA and p53 (data not shown).

In order to monitor keratinocyte differentiation in the tumours, the expression of epithelial syndecan-1, which is known to decrease during tumorigenesis, was analysed in the same skin lesions via immunoperoxidase staining. Syndecan-1, like Mad, was most abundantly expressed in the differentiating suprabasal layers of epidermis and in the dermal hair follicle cysts, whereas the dermis was negative. No specific signal was observed in the sections stained with control antibody (data not shown). Hyperplastic DMBA-treated epidermis showing elongated rete ridges, exhibited distinct syndecan-1 staining that was slightly discontinuous and irregular, whereas the basal cell layer and the superficial differentiated epidermal cell layer were negative (Figure 5a and b). In dysplastic areas, atypical keratinocytes showed intensive staining, while the basal cell layer was negative (Figure 5c). Some syndecan-1 expression was seen, in the well-differentiated areas of the squamous cell carcinomas where keratin horn cysts were formed (Figure 5c), whereas the expression was lost in the poorly differentiated areas of these tumours (Figure 5d).

*Mad expression in human squamous cell carcinoma, basal cell carcinoma and melanoma*

The human squamous cell carcinoma biopsies showed hyperkeratosis, parakeratosis, hyperplastic epidermis with cell dysplasia and various degrees of differentiation including horn cyst formation and invasion in the dermis. Strong Mad signals were detected in the thickened stratum granulosum and in the cells around the horn cysts (Figure 6a and b). Superficial spreading-type basal cell carcinomas showed distinct Mad expression in the stratum granulosum but the peripheral palisading basal carcinoma cells were negative. Autoradiographic grains were present in keratotic basal cell carcinomas forming horn cysts and also in the cells surrounding these cysts, which are considered to represent incomplete hair shaft formation (Figure 6c and d). No Mad expression was detected in nodular melanoma (data not shown). The biopsies also contained areas of adjacent normal healthy skin, where the Mad expression pattern was observed to be similar to that of normal adult mouse skin (Figure 6e and f). Mad expression was also noted inside the sebaceous glands and in the keratinocytes surrounding the lumen of the hair shafts (data not shown). Sections of normal human skin hybridised with the Mad sense strand gave only background unspecific signal from the most superficial keratin layers (inset Figure 6e).

**Figure 5** Immunostaining for syndecan-1 in carcinogen-treated skin and carcinogen-induced skin tumours. (**a** and **b**) Distinct syndecan-1 staining in the suprabasal epidermal layers in hyperplastic epidermis. In the horn cysts in well-differentiated squamous cell carcinoma (**c**), syndecan-1 staining is decreased and restricted to the differentiating cells, whereas in the dysplastic areas only very weak staining can be detected. Epidermal cells (**d**) stain positively for syndecan-1 while the undifferentiated anaplastic squamous cell carcinoma cells in the dermis are negative. The lesions stained were obtained from the same samples as those shown in Figure 4. Bar = 0.1 mm.

**Figure 6** Mad expression in human squamous and basal cell carcinomas. (**a** and **b**) Intensive Mad signal in the stratum granulosum in a squamous cell carcinoma and in the cells that form a horn cyst, which is seen only partially in the lower part of the figure (arrows in **a** and **b**). In the upper layers of the skin hyperkeratosis, parakeratosis and necrotic keratinocytes can be detected (arrowheads in **a** and **b**; the skin surface is just beyond the upper margin of the figure). (**c** and **d**) A keratotic basal cell carcinoma forming horn cysts. Strong Mad signal is found in the keratinocytes surrounding the horn cyst (arrow). (**e** and **f**) Hybridisations of normal human adult skin with the Mad antisense probe; the inset in **e** shows control hybridisation with the Mad sense probe. Bar = 0.1 mm.

### Discussion

In this study we have observed that Mad mRNA is highly expressed in differentiating epidermal keratinocytes in normal epidermis, healing skin wounds and epidermal tumours, whereas the proliferating basal epidermal cells are negative. This localised expression supports the hypothesis that Mad is associated with cell differentiation. Analogous results have been published for leukaemia cell lines differentiating in culture (Ayer and Eisenman, 1993; Larsson *et al.*, 1994; Zervos *et al.*, 1993). However, unlike leukaemia cells in culture, the proliferating and differentiating epidermal keratinocytes expressed little or no c-Myc, suggesting that steady-state c-Myc mRNA levels, even in proliferating cells, are significantly lower than Mad mRNA levels in the differentiating cells. This result is of interest, as Mad is currently held to lack intrinsic functional activity and believed to function only as an antagonist of Myc and a repressor of Myc-regulated genes in a ternary complex with Max and the mammalian homologues of the yeast repressor Sin3 (Ayer *et al.*, 1995). Clearly, other members of the *myc* gene family may interact with Mad in the epidermal keratinocytes. It should also be mentioned that two recently discovered Max-interacting bHLHZip proteins, Mad3 and

Mad4, are related to Mad and Mxi1 and that the heterocomplexes Mad3–Max, Mad4–Max repress transcription by binding to the same E-box-like sequences that mediate Myc–Max activation (Hurlin *et al.*, 1995).

Differentiating cells in hyperplastic lesions and cells surrounding horn cysts in well-differentiated squamous cell carcinomas strongly expressed Mad mRNA. Dysplastic epidermal cells, although benign and with a preserved basement membrane, expressed only low levels of Mad. Mad was therefore observed in benign as well as in malignant lesions. A few of the most malignant lesions that had lost the structure of epidermal stratification and differentiation were negative for Mad. However, even in the presence of a Mad mRNA signal, the possibility remains that the *mad* gene has suffered small mutations in critical regions, for example in the region encoding the 25 first amino acid residues, which are important for its function as a transcriptional repressor (Ayer *et al.*, 1995) and presumably as an inhibitor of cell growth (Västrik *et al.*, 1995).

The expression pattern of Mad was different from that of syndecan-1, which began in a deeper epidermal layer and also extended to the rete ridges. However, there was significant correlation of Mad and syndecan-1 expression in the skin tumours in relation to their degree of malignancy. Syndecan-1 is known to be down-regulated in the most malignant skin lesions (Inki *et al.*, 1994), possibly owing to the loss of epidermal layered cytoarchitecture. Thus, syndecan-1 provides a marker for the loss of epidermal cell differentiation associated with malignant progression, and our studies show that the expression of this marker is correlated well with that of Mad. interestingly, the syndecan-1 gene promoter also contains several Myc target sequences (Hinkes *et al.*, 1993), which may be regulated by members of the Myc oncoprotein transcription factor family.

These studies indicate that Mad mRNA expression is associated with epithelial keratinocyte differentiation, but that it is not expressed in rapidly dividing basal epidermal cells. Furthermore, Mad expression is up-regulated in hyperproliferative epidermis, increasing with the thickness of the stratum granulosum. Our results also suggest that Mad expression can occur in both benign and malignant hyperproliferative lesions as long as the cells retain differentiation potential. Loss of expression was seen only in the most anaplastic areas of the tumours. A similar expression pattern was evident in murine and human skin and in chemically induced and naturally occurring skin tumours, suggesting an important role for this transcription factor in the regulation of keratinocyte differentiation.

**Acknowledgements**
We thank Dr Kari Alitalo for encouraging discussions and Dr Jorma Keski-Oja for thoughtful comments on the manuscript. We also thank Kirsti Tuominen, Tapio Tainola, Raili Taavela and Mari Helanterä for excellent technical assistance. This study was supported by the Finnish Cancer Organizations, the Finnish Academy, the Ida Montins Foundation and the Sigrid Juselius Foundation.

### References

ALITALO K, MÄKELÄ TP, SAKSELA K, KOSKINEN P AND HIRVONEN H. (1992). Oncogene amplification: analysis of *myc* oncoproteins. In *Gene Amplification in Mammalian Cells: Techniques and Applications*, R Kellems (ed.), pp. 371–382. Marcel Dekker: New York.

AMATI B, LITTLEWOOD TD, EVAN GI AND LAND H. (1993). The c-Myc protein induces cell cycle progression and apoptosis through dimerization with Max. *EMBO J.*, **12,** 5083–5087.

AYER DE AND EISENMAN RN. (1993). A switch from Myc:Max to Mad:Max heterocomplexes accompanies monocyte/macrophage differentiation. *Genes Dev.*, **7,** 2110–2119.

AYER DE, KRETZNER L AND EISENMAN RN. (1993). Mad: A heterodimeric partner for Max that antagonizes Myc transcriptional activity. *Cell*, **72,** 1–20.

AYER DE, LAWRENCE QA AND EISENMAN RN. (1995). Mad–Max transcriptional repression is mediated by ternary complex formation with mammalian homologs of yeast repressor Sin3. *Cell*, **80,** 1–20.

BJELOGRLIC NM, MÄKINEN M, STENBÄCK F AND VÄHÄKANGAS K. (1994). Benzo[a]pyrene-7,8-diol-9,10-epoxide-DNA adducts and increased p53 protein in mouse skin. *Carcinogenesis*, **15,** 771–774.

BLACKWOOD EM AND EISENMAN RN. (1991). Max: a helix–loop–helix zipper protein that forms a sequence-specific DNA-binding complex with myc. *Science*, **251,** 1211–1217.

BREMNER R, KEMP CJ AND BALMAIN A. (1994). Induction of different genetic changes by different classes of chemical carcinogens during progression of mouse skin tumors. *Mol. Carcinog.*, **11,** 90–97.

CANTLEY LC, CARPENETER C, DUCKWORTH B, GRAZIANI A, KAPELLER R AND SOLTOFF S. (1991). Oncogenes and signal transduction. *Cell*, **64,** 281–302.

CHOMCZYNSKI P AND SACCHI N. (1987). Single-step method of RNA isolation by acid guanidinium thiocyanate–phenol–chloroform extraction. *Anal. Biochem.*, **162,** 156–159.

ELENIUS K, VAINIO S, LAATO M, SALMIVIRTA M, THESLEFF I AND JALKANEN M. (1991). Induced expression of syndecan in healing wounds. *J. Cell Biol.*, **114,** 585–595.

FUCHS E. (1990). Epidermal differentiation: the bare essentials. *J. Cell Biol.*, **111,** 2807–2814.

FUCHS E. (1993). Epidermal differentiation and keratin gene expression. *J. Cell Sci. Suppl.*, **17,** 197–208.

HINKES MT, GOLDBERGER OA, NEUMANN PE, KOKENYESI R AND BERNFIELD M. (1993). Organization and promoter activity of the mouse syndecan-1 gene. *J. Biol. Chem.*, **268,** 11440–11448.

HURLIN PJ, QUEVA C, KOSKINEN PJ, STEINGRIMSSON E, AYER DE, COPELAND NG, JENKINS NA AND EISENMAN RN. (1995). Mad3 and Mad4: novel Max-interacting transcriptional repressors that suppress c-myc dependent transformation and are expressed during neural and epidermal differentiation. *EMBO J.*, **14,** 5646–5659.

INKI P, JOENSUU H, GRENMAN R, KLEMI P AND JALKANEN M. (1994). Association between syndecan-1 expression and clinical outcome in squamous cell carcinoma of the head and neck. *Br. J. Cancer*, **70,** 319–323.

JALKANEN M, NGUYEN H, RAPRAEGER A, KURN N AND BERNFIELD M. (1985). Heparan sulfate proteoglycans from mouse mammary epithelial cells: localization on the cell surface with a monoclonal antibody. *J. Cell Biol.*, **101,** 976–984.

KORHONEN M AND STENBACK F. (1984). Adenocarcinoma metastatic to the uterine cervix. *Gynecol. Obstet. Invest.*, **17,** 57–65.

LARSSON L, PETTERSSON M, ÖBERG F, NILSSON K AND LÜSCHER B. (1994). Expression of *mad*, *mxi1*, *max* and c-*myc* during induced differentiation of hematopoietic cells: opposite regulation of *mad* and c-*myc*. *Oncogene*, **9,** 1247–1252.

LÜSCHER B AND EISENMAN RN. (1990). New light on Myc and Myb. Part I. Myc. *Genes Dev.*, **4,** 2025–2035.

MALI M, JAAKKOLA P, ARVILOMMI AM AND JALKANEN M. (1990). Sequence of human syndecan indicates a novel gene family of integral membrane proteoglycans. *J. Biol. Chem.*, **265,** 6884–6889.

MORGENBESSER SD AND DEPINHO RA. (1994). Use of transgenic mice to study myc family gene function in normal mammalian development and in cancer. *Cancer Biol.*, **5,** 21–36.

STENBÄCK F. (1978). Life history and histopathology of ultraviolet light-induced skin tumors. *Natl Cancer Inst. Monogr.*, **50,** 57–70.

TOKUNAGA K, TANIGUCHI H, YODA K, SHIMIZU M AND SAKIYAMA S. (1986). Nucleotide sequence of a full-length cDNA for mouse cytoskeletal beta-actin mRNA. *Nucleic Acids Res.*, **14,** 2829–2833.

VÄSTRIK I, KOSKINEN PJ, ALITALO R AND MÄKELÄ TP. (1993). Alternative mRNA forms and open reading frames of the *max* gene. *Oncogene*, **8,** 503–507.

VÄSTRIK I, KAIPAINEN A, PENTTILÄ TL, LYMBOUSSAKI A, ALITALO R, PARVINEN M AND ALITALO K. (1995). Expression of the Mad gene during cell differentiation *in vivo* and its inhibition of cell growth *in vitro*. *J. Cell Biol.*, **128,** 1197–1208.

WERNER S, PETERS KG, LONGAKER MT, FULLER-PAGE F, BANDA MJ AND WILLIAMS LT. (1992). Large induction of keratinocyte growth factor expression in the dermis during wound healing. *Proc. Natl Acad. Sci. USA*, **89**, 6896–6900.

WILKINSON DG, BAILES JA, CHAMPION JE AND MACMAHON AP. (1987*a*). A molecular analysis of mouse development from 8 to 10 days post coitum detects changes only in embryonic globin expression. *Development*, **99**, 493–500.

WILKINSON DG, BAILES JA AND MACMAHON AP. (1987*b*). Expression of proto-oncogene int-1 is restricted to specific neural cells in the development of mouse embryos. *Cell*, **50**, 79–88.

YUSPA SH. (1994). The pathogenesis of squamous cell cancer: Lessons learned from studies of skin carcinogenesis. Thirty-third GHA Clowes memorial award lecture. *Cancer Res.*, **54**, 1178–1189.

YUSPA SH, KILKENNY A, CHENG C, ROOP D, HENNINGS H, KRUSZEWSKI F AND LEE E. (1991). Alterations in epidermal biochemistry as a consequence of stage-specific genetic changes in skin carcinogenesis. *Environ. Health Perspect.*, **93**, 3–10.

ZERVOS A, GYURIS J AND BRENT R. (1993). Mxi1, a protein that specifically interacts with Max to bind Myc–Max recognition sites. *Cell*, **72**, 223–232.